# The Influence of Trait Emotion and Spatial Distance on Risky Choice Under the Framework of Gain and Loss

**DOI:** 10.3389/fpsyg.2022.592584

**Published:** 2022-06-03

**Authors:** Fuming Xu, Long Huang

**Affiliations:** ^1^School of Education Science, Nanning Normal University, Nanning, China; ^2^School of Psychology, Jiangxi Normal University, Nanchang, China; ^3^School of Humanities and Management, Wannan Medical College, Wuhu, China

**Keywords:** framing effect, psychological distance, trait anxiety, trait anger, risky choice, Asian Disease Problem

## Abstract

In the time of the COVID-19 pandemic, people are often faced with uncertain risky choice. Risky choice will be affected by different descriptions of the event’s gain or loss framework, this phenomenon is known as the framing effect. With the continuous expansion and in-depth study of frame effects in the field of risky choice, researchers have found that the are quite different in different situations. People have different interpretations of the same event at different psychological distances, and will also be affected by their own emotions. Therefore, the current study examines the common influence of task frame, spatial distance, and trait emotion on risky choice through two studies. Study 1 used a 2 (framework: gain vs. loss) × 2 (trait sentiment: high vs. low) inter-subject design, and the dependent variable is the choice of the rescue plan for the classic “Asian disease” problem. The results revealed that trait anger did not predict individuals’ risky choice preferences, and high trait anxiety led individuals to be more risk-averse. The framing effect exists in risky choice, and individuals prefer risk seeking in the loss frame. Study 2 used a 2 (spatial distance: distant vs. proximal) × 2 (framework: gain vs. loss) × 2 (trait sentiment: high vs. low) three-factor inter-subject design in which the dependent variable is the choice of rescue plan. The results indicate that the framing effect also exists in risky choice, and individuals prefer risk seeking in a loss frame. High trait anxiety lead individuals to be more risk-averse, while trait anger has no significant predictive effect on risk preference. Distant spatial distance lead individuals to increase their preference for risk-seeking under the gain frame, which leads to the disappearance of the framing effect. In conclusion, trait anxiety and spatial distance have a certain degree of influence on risky choice under the framework of gain and loss.

## Introduction

In the time of the COVID-19 pandemic, people are often faced with uncertain risky choice. Risky choice will be affected by different descriptions of the event’s gain or loss framework, this phenomenon is known as the framing effect. The framing effect is the core theme of behavioral decision-making research and one of the most universal discoveries in the field of judgment and decision-making. Kahneman and Tversky (1979) first discovered the existence of the framing effect in their study on the “Asian Disease Problem.” In this study, the “Asian Disease Problem” assumes that the disease has the potential to kill 600 people, and the decision-makers face two different frames of choice. In the first (gain) frame, the decision-makers can choose between option A (200 people saved) and option B (there is a 1/3 probability that 600 people will be rescued and a 2/3 probability that no one will be rescued). In the second (loss) frame, the decision-makers can choose between option C (400 deaths) and Scenario D (there is a 1/3 probability that no one will die and a 2/3 probability that 600 people will die). This is a typical example of framing effect in risky choice (Tversky and Kahneman, 1981), where the alternatives under the two frames express exactly the same content, but the way of expressing them has changed, resulting in a change in the decision-maker’s choice preference. People tend to choose option A (risk aversion) under the first frame and option D (risk-seeking) under the second frame. The phenomenon that the choice preference changes due to the change of the situational expression is called the framing effect. Kahneman and Tversky (1982) explained the framing effect based on the mechanism of gains and losses reference points. They believed that when faced with the gain frame, the decision-makers would make a “gain” valuation of the profit and loss situation relative to the “lost” reference point. Under such circumstances, the decision-making behavior tends to be risk-averse and to conservatively choose certain options. However, when faced with the loss frame, the decision-makers will make a “lost” valuation of the profit and loss situation relative to the “gain” reference point, resulting in a tendency for risk-seeking behavior, and taking risks to choose uncertain options. The discovery of the framing effect has aroused the general attention and interest of researchers in the fields of psychology and behavioral economics. Subsequent researchers began to conduct diversified operations on the independent variable decision frame, the probability level in the research paradigm, the amount of profit and loss, and the content of the decision. The results of a series of confirmatory studies confirmed the influence of the framing effect on risky choice (Wang, 1996; Kühberger, 1998; Levin et al., 2002; Maule and Villejoubert, 2007a). The framing effect starts from the cognitive process of risk perception, cognitive processing, and profit and loss calculation of decision-making information. The size of the framing effect is affected by factors such as the cognition and emotion of the decision-maker (Maule and Villejoubert, 2007b; Seta et al., 2017).

### Framing Effect and Emotion

Although the cognitive dual-system theory believes that emotions can help decision-makers make quick and accurate judgments and decisions, emotions can also interfere with their rational judgments and bias information retrieval and cognition. In the study of emotional intensity, researchers found that people with stronger emotions rely more on intuition and emotional inspiration, leading to risk aversion under the income framework and risk-seeking under the loss framework, resulting in a clear framing effect. In contrast, people who are lesser “emotional” are more dependent on analysis and careful consideration and prefer risk-neutral behaviors (Slovic and Peters, 2006). In the research of emotion types, most are driven by valence theory (Forgas, 1995), which believes that negative emotions can lead to relatively pessimistic expectations, and thereby risk aversion decisions. On the other hand, positive emotions can lead to relatively optimistic expectations and thereby risk-seeking decisions (Mayer and Hanson, 1995; Waters, 2008). It can be seen that anxiety and fear as negative emotions may lead to risk aversion in decision-making. However, recent research refutes that all negative emotions conform to the risk aversion hypothesis of valence theory (Lerner and Tiedens, 2006). For example, anger and anxiety, which this study focuses on, are two different types of negative emotions. They are both stable negative personality traits. Under the same conditions, individuals with high trait anger were more likely to express anger than those with low trait anger. Individuals with high trait anxiety are more likely to exhibit signs of anxiety than individuals with low trait anxiety (Endler and Kocovski, 2001). But the functions of their neurobiological systems are pretty differentiated. Anxiety is a negative emotional trait expressed by the activity of a behavioral inhibition system (BIS) that regulates passive-avoidance behavior and is sensitive to punishment and dependent on serotoninergic transmission (Rex and Fink, 2011). The anxiety system controls reactions that facilitate a defensive approach (resolve approach-avoidance conflicts). Anxiety is susceptible to defensive distance (Mcnaughton and Corr, 2004), whereas anger relates to an appetitive or approach motivational system expressed by a behavioral approach system (BAS), and is mainly served by the noradrenergic transmission Eddie (2003). Therefore, the effects of trait anxiety and trait anger on individual risky choice may be different or even opposite. Lerner and Keltner’s (2001) study found that although anxiety and anger have the same valence, they will produce opposite effects in a risky decision. The uncertainty and lack of control associated with anxiety lead to the gains and losses of individuals with high trait anxiety. Risk aversion choices are made in the framework; the certainty and sense of control associated with anger will cause high-quality angry individuals to make risk-seeking choices in the framework of gains and losses, and this influence remains stable under different framework conditions (Lerner and Keltner, 2001). However, most of the existing studies are carried out in western countries, and there are few studies on the effect of specific emotions on the framing effect in risky choice in China. China’s cultural background is different from that of western countries. Research has found that in western countries dominated by individualistic culture, people are relatively comfortable in expressing their negative emotions, while in China, dominated by collectivist culture, people may feel ashamed to express their negative emotions (Chen, 2011), especially anger. In western cultures, people prefer to express their anger, while in eastern cultures people prefer to suppress this emotion. People suppress anger to avoid its potential impact on them, which is in line with the requirements of Chinese Confucianism for “self-denial and return to courtesy” in behavior. Based on cultural considerations, Americans may be affected by anger when making risky decisions, while Chinese people may not. Similarly, people in China may inhibit their expression of anxiety, and the inhibition of expression will reduce their risk-taking behavior (Li et al., 2015). However, with the integration of eastern and western cultures in recent years, young people in China have been greatly influenced by western culture. Therefore, the influence of idiosyncratic sentiment on the framing effect in risky choice in China is still unclear. Based on this, this study explores the influence of trait anxiety and trait anger on the framing effect in risky choice in the cultural background of China, and proposes the following hypothesis:

Hypothesis 1: Trait emotions (trait anxiety and trait anger) influence the risk choice framing effect.

### Framing Effect and Psychological Distance

With the continuous expansion and in-depth study of framing effects in the field of risky choice, researchers have found that its effects are quite different in different situations. For example, Li et al. (2000) did not observe obvious framing effects in the context of the gas explosion problem. This may be because there is no coal mine in the location of the subject, so it is difficult for the decision-maker to envisage such a situation. The long-space distance leads to the disappearance of the framing effect. However, Tversky and Kahneman (1981) first discovered the framing effect in the Asian Disease Problem context. This may be because this problem needs to be faced across the country, so it is close to the decision-makers, which leads to the framing effect. It is speculated that the spatial distance between the event and the decision-maker may have an impact on people’s information processing and risk perception, which in turn affects the framing effect of decision-making. Spatial distance is a type of psychological distance. Trope and Liberman (2003) first proposed the concept of psychological distance, including time, spatial, social, and probability distance. In recent years, researchers have carried out a series of empirical studies on the effect of psychological distance on the framing effect in risky choice. For example, McElroy and Mascari (2007) explored the influence of time distance on the framing effect in risky choice and found that the effect is not obvious when the time distance is proximal. Participants prefer risk aversion regardless of the framework and the framing effect is enhanced at a long distance. Zhong et al. (2009) used a sample of Chinese college students to verify McElroy and Mascari (2007)’s research conclusions. Duan et al. (2013) comprehensively explored the influence of psychological distance in four dimensions on the risk selection framing effect and found that subjects with long psychological distance produced a more obvious framing effect, while that of proximal psychological distance was significantly weakened. On the contrary, Raue et al. (2015) explored the influence of psychological distance and decision-making framework on risk preference but found that the influence of psychological distance on risk preference only exists in the acquisition frame. In the loss frame, participants prefer risk-seeking. The authors found that while the framing effect only occurs in the context of close psychological distance, the effect weakens or even disappears in the context of long psychological distance, which is contrary to the aforementioned research conclusions (Raue et al., 2015). Sun et al. (2017) findings support the weakening of the framing effect of psychological distance. They found that in the context of obtaining the frame, the farther the social distance, the more risk-neutral individual decision-making is, which indicates that the increase in social distance weakens the benefit of the framing effect. Similarly, Zhang et al. (2017) also found that in the benefit situation, the increase of social distance will lead to an increase in individual risk-seeking, and proximal social distance tends to be more risk-averse than distant social distance. In the loss scenario, the increase in the social distance leads to a decrease in individual risk-seeking. Proximal social distance is more risk-seeking than distant social distance, that is to say, social distance will weaken the framing effect in risky choice. In general, the research results on the framing effect of psychological distance have not yet reached a consistent conclusion, and there are relatively few studies on the effect of spatial distance on the framing effect of risky choice in the existing literature. With the rapid development of science and technology, remote decision-making has become an important part of daily work and life in modern society. People are often faced with decision-making on foreign investment projects and the division of foreign tasks. Whether there is a difference between the risky choice of events that occurred in other places and this place, and the influence of spatial distance on the effect of the framing effect in risky choice deserves further study. Based on this, the research proposes the following hypothesis:

Hypothesis 2: Spatial distance influences the framing effect in risky choice.

Combining the aforementioned arguments and hypotheses about the influence of trait emotions on the framing effect in risky choice, the current study formulates the following hypothesis:

Hypothesis 3: Spatial distance and trait emotions jointly influence the risk selection framing effect.

## Study 1

### Design and Subjects

A between-subjects 2 (frame: gain vs. loss) **×** 2 (trait sentiment: high vs. low) design was adopted, and the dependent variable was the choice of two rescue options.

Following previous research (Lerner and Keltner, 2001), we chose the medium effect size (0.15) as the standard. With an alpha of 0.05 and Power = 0.95, the projected sample size needed to obtain the effect was *N* = 107. To obtain this sample size, a total of 134 undergraduates from a university (30 males and 104 females) were recruited, with an average age of 20.56 ± 0.95 years. Participants were randomly assigned to two decision-making situations: gain and loss. Among them, a total of 62 subjects were in the gain frame group, and a total of 72 subjects were in the loss frame group.

### Procedure

Subjects were told that they were participating in a study on risky choice. They first completed the trait anxiety and trait anger scales and then completed the risky choice context questionnaire. Finally, they completed questions on general demographics, including gender and age.

### Materials

#### Risk Selection Questionnaire

The risk selection questionnaire was adapted from Tversky and Kahneman’s (1981) “Asian Disease Problem.” The phrase was changed from “Asian Disease” to “African Disease,” and the other contents are consistent with the original situation. As follows:

Imagine that China is preparing for the outbreak of an unusual African disease, which is expected to kill 600 people. Two alternative programs to combat the disease have been proposed. Assume that the exact scientific estimate of the consequences of the programs is as follows:

A: If Program A is adopted, 200 people will be saved.
B: If Program B is adopted, there is a 1/3 probability that 600 people will be saved, and a 2/3 probability that no people will be saved.

Which of the two programs would you favor?

For the second group of subjects (Group 2), instead of Programs A and B, the following alternative Programs C and D were given (all else the same):

C: If Program C is adopted, 400 people will die.
D: If Program D is adopted, there is a 1/3 probability that nobody will die, and a 2/3 probability that 600 people will die.

Which of the two programs would you favor?

#### Trait Anxiety Scale

The trait anxiety part of the Chinese version of the State-Trait Anxiety Inventory (STAI) compiled by Spielberger (2010), translated and revised by Zheng and Li (1997), was used to measure the subjects’ trait anxiety. There are a total of 20 items on this four-point scale and the higher the score, the more obvious the tendency of trait anxiety. After testing, the internal consistency coefficient of the scale in the current study is 0.88.

#### Trait Anger Scale

The trait anger scale (TAS) compiled by Spielberger and Reheiser (2010) was used to measure the trait anger of the subjects. There are a total of 10 items on this four-point scale. The higher the score, the more obvious the tendency to trait anger. After testing, the internal consistency coefficient of the scale is 0.80.

## Results

Regarding the research method of Lerner and Keltner (2001), we followed Judd and McClelland (1989) procedure for mixed design regression. The first model regressed the average of the respondents’ preferences on the decision-making frame, taking risk selection as the dependent variable. It was found that there was a significant framing effect (OR = 1.24, *p* < 0.01).

The second model regressed the average of the respondents’ preferences on trait emotion. It was found that trait anxiety negatively predicted participants’ risk-seeking and positively predicted participants’ risk aversion (OR = −2.45, *p <* 0.001). Participants who reported higher trait anxiety scores were more reluctant to choose risky options in risky decisions. However, trait anger could not predict participants’ risk preference (OR = 0.50, *p* > 0.05). Since trait anger has no significant predictive power, we only analyze trait anxiety in the following analyses.

**FIGURE 1:**
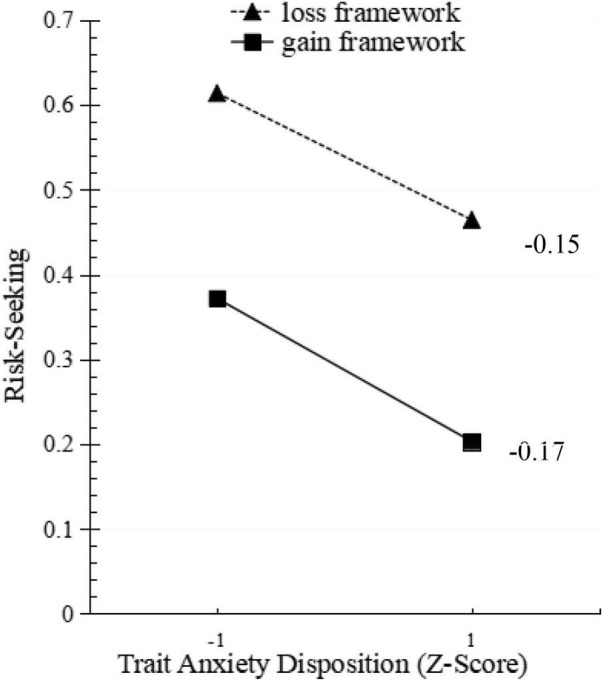
The effects of trait anxiety and framing effects on risk-seeking.

The third model regressed the respondents’ preferences on the explanatory variables: decision-making framework, trait anxiety, and the interaction between trait anxiety and decision frame. It was found that trait anxiety still negatively predicted participants’ risk-seeking and positively predicted participants’ risk aversion (OR = −2.12, *p* < 0.01). Participants who reported higher trait anxiety scores were more reluctant to choose risky options in risky decisions. The framing effect is no longer significant (OR = 0.46, *p* > 0.05). The interaction between decision frame and trait anxiety was not significant (OR = 0.30, *p* > 0.05). An exploratory examination of these patterns within each frame (see Figure 1) does, indeed, trait anxiety has no apparent effect on the framing effect of risky decision-making.

So, it can be seen that trait anxiety can affect risk preference in decision-making; but trait anger cannot affect risk preference in decision-making. Moreover, trait anxiety and trait anger have not been found to have an impact on the framing effect in risky choice. Therefore, Hypothesis 1 is partially supported.

Study 1 found that there is a significant framing effect in risky choice that do not emphasize spatial distance. The acquisition frame group is more inclined to risk aversion, and the loss frame group is more inclined to risk-seeking. In addition, trait anger does not have a significant impact on the framing effect of risk decision-making, only trait anxiety has an impact on risk preference. This result is different from that of Lerner and Keltner (2001). This may be because it is affected by the subjects’ cultural background, perception of spatial distance, and other factors. Therefore, based on Study 1, this study further increases the variable of spatial distance to explore the common influence of trait emotion and spatial distance on the risk selection framing effect.

## Study 2

### Design and Subjects

Study 2 uses a 2 (spatial distance: distant vs. proximal) × 2 (decision framework: gain vs. loss) × 2 (trait sentiment: high vs. low) three-factor between-subjects design. The dependent variable is the choice of either of two rescue plans. We chose the medium effect size (0.15) as the standard. With an alpha of 0.05 and Power = 0.95, the projected sample size needed to obtain the effect was *N* = 119. To obtain this sample size, a total of 160 undergraduates from a university (42 males and 118 females) were recruited to participate in Study 2. Participants were randomly assigned to four decision-making situations, including a proximal-space distance-acquisition group with 37 people, proximal-space distance-loss group with 41 people, long-space distance-acquisition group with 39 people, and long-space distance-loss group with 43 people. None of the subjects had participated in similar studies before.

### Procedure

Same as Study 1.

### Materials

#### Situation Questionnaire

We referred to Tversky and Kahneman’s (1981) “Asian Disease Problem,” compiled the “Biochemical Weapons Problem,” and embedded the accident location as “H 2,000/200 km away” in the description of the decision-making situation. “City” is a spatial distance variable. See Appendix 1 for the specific situation. Before the formal study, 220 college students were randomly selected, and they were asked to rate from 1 to 4 (impossible to completely possible) whether the situational questionnaire might happen and use the 7-point scoring system to self-evaluate their feelings under the two-distance situations. The psychological distance value of the larger the value, the farther the psychological distance was used to test the validity of the problem situation and the validity of the spatial distance operation. The results indicated that the adaptation scenario is a realistic situation that may occur (*M* = 3.02). The difference in the psychological distance self-evaluation scores felt by different spatial distance scenarios is statistically significant (*t* = −6.84, *p* < 0.001, Cohen’s *d* = 2.45). The psychological distance felt by the subjects in the scenario of “2,000 kilometers away” was greater than that in the scenario of “200 kilometers away.” This shows that the operation of the problem situation and spatial distance in this research is effective.

#### Trait Anxiety Scale and Trait Anger Scale

As in Study 1, the internal consistency coefficients of the two scales in Study 2 were 0.90 and 0.81, respectively.

## Results

With reference to the research method of Lerner and Keltner (2001), we followed Judd and McClelland (1989) procedure for mixed design regression. The first model regressed the average of the respondents’ preferences on the decision-making frame, the spatial distance, and the interaction terms of the decision frame and the spatial distance, taking risk selection as the dependent variable. It was found that there was a significant framing effect (OR = −1.14, *p* < 0.05). The predictive effect of spatial distance was not significant (OR = −0.21, *p* > 0.05). The interaction effect of decision frame and spatial distance was significant (OR = 1.27, *p* < 0.05). The selection of subjects in different spatial distance groups under the acquisition and loss frameworks was further tested, and it was found that under the former, the choices of subjects in different spatial distance groups were significantly different [χ^2^(1) = 5.38, *p* = 0.02, φ = 0.27], the distant-space distance group prefers risk-seeking than the proximal-space distance group. However, under the loss framework, there is no significant difference in the risk choices of subjects in different spatial distance groups [χ^2^(1) = 0.23, *p* = 0.63, φ = −0.05]. Hypothesis 2 is verified.

The second model regressed the average of the respondents’ preferences on trait emotion. It was found that trait anxiety negatively predicted participants’ risk-seeking and positively predicted participants’ risk aversion (OR = 0.95, *p* < 0.05). Participants who reported higher trait anxiety scores were more reluctant to choose risky options in risky decisions. However, trait anger only marginally significantly predicted participants’ risk preference (OR = 1.09, *p* < 0.01). Moreover, trait anger predicted risky decision-making in the opposite direction than trait anxiety. Since trait anger has no significant predictive power, we only analyze trait anxiety in the following analyses.

**FIGURE 2:**
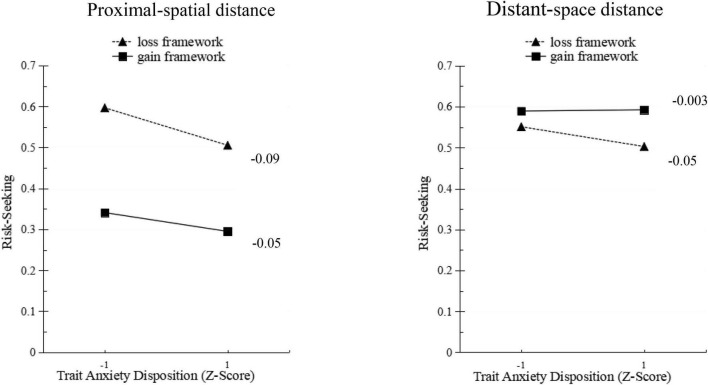
The impact of framing effect and trait anxiety on risk preference under different spatial distance conditions.

The third model regressed the respondents’ preferences on the explanatory variables: decision-making framework, the spatial distance, trait anxiety, and the interaction between these three variables. It was found that the predictive effect of the decision framework was still significant (OR = −1.08, *p* < 0.05). The main effect of spatial distance was not significant (OR = −0.20, *p* > 0.05). The predictive effect of trait anxiety was also not significant (OR = −0.03, *p* > 0.05). The predictive effect of the interaction term of the three variables was significant (OR = 0.03, *p* < 0.05). An exploratory examination of these patterns within each spatial distance (see Figure 2) does. Relative to proximal spatial distance, distant-spatial distance increased participants’ risk-seeking under the acquisition frame, resulting in the disappearance of the framing effect in distant-spatial distance. In the close spatial distance, trait anxiety has a greater effect on risk-seeking in the loss framework than in the gain framework. High trait anxiety reduced participants’ appetite for risk under the loss framework. At this time, the framing effect in risky choice. Therefore, trait anxiety and spatial distance have a certain impact on framing effects in risky choice, which does support Hypotheses 1 and 3.

## Discussion

Both Study 1 and Study 2 found that trait anxiety had a significant impact on risky choice. Individuals with high trait anxiety were more risk-averse, and individuals with low trait anxiety were more risk-seeking. While trait anger was not found to have a significant effect on risky choice, only marginally significant was found in Study 2. Moreover, trait anger predicts risky decisions in the opposite direction as trait anxiety predicts risky decisions. The revised Reinforcement Sensitivity Theory (RST) can explain this phenomenon. Revised Reinforcement Sensitivity Theory believes that although fear and anxiety are both negative emotions, they are distinct and in a sense opposite. They are controlled by interacting separate brain systems (Gray and McNaughton, 2000; Mcnaughton and Corr, 2008). Revised RST differentiates human behavior based on motivational factors. Negative emotions (e.g., anxiety) are associated with avoidance motivation, while anger violates this rule. Anger like most positive emotions is associated with approach motivation (Moons et al., 2010). Thus, compared to anxiety, anger typically provokes greater motivation to act using the behavioral approach system (BAS), leading to more risky decisions (Smith et al., 2008). Anxiety uses the behavioral inhibition system (BIS) to act, prompting people to be cautious and avoid harm, leading to more risk aversion (Perkins et al., 2007; Wu et al., 2018).

The Assessment Tendency Framework (ATF) proposed by Lerner et al. (2003) can also explain the different predictions of anger and anxiety on risky decision-making. ATF believes that anxiety and anger are both negative emotions, but differ in the dimensions of certainty and controlling evaluations. Anxiety is low certainty and low control. Individuals who feel anxiety perceive higher risk, leading individuals to tend to overestimate risk in decision-making. And anger is high certainty and high control. Individuals who feel angry perceive lower risk, leading individuals to tend to underestimate risk in decision-making. Thus, anxiety and anger affect decision-making in different directions (Lerner et al., 2003). This is consistent with previous research conclusions (Lerner and Keltner, 2001; Peng et al., 2014). Furthermore, no significant effect of trait anger on risky choice was found in this study, which may also be because the Chinese, who are dominated by collectivist cultures, are shy about expressing their negative emotions. Chinese in particular refrain from expressing outwardly directed emotions such as anger (Chen, 2011). Chinese prefer to restrain their anger in line with Chinese Confucianism for “self-denial and return to courtesy” in behavior. This result is also consistent with the findings of She et al. (2017) study of Chinese subjects.

This study systematically and integratedly explored the spatial distance, decision-making framework, and idiosyncratic emotions in risk selection. The results revealed that the main effect of spatial distance is significant, the subjects’ choice preference is risk avoidance under the condition of proximal spatial distance, and risk-seeking under the condition of long spatial distance, which are consistent with most previous research conclusions on other dimensions of psychological distance (McElroy and Mascari, 2007; Zhong et al., 2009; Chen and He, 2014). Construal level theory believes that people tend to form abstract, simple, and de-contextualized high-level representations of things that are far away in the psychological distance, and low-level concrete, complex, and contextualized things that are closer in the psychological distance. A low level of explanation will divert the decision-maker’s attention to the feasibility of the result, while a high level of abstract explanation will divert attention to the value of the result. Therefore, as the psychological distance increases, the feasibility of the results closely associated with a low level of interpretation has less impact on decision-making, while the desirability of results closely associated with a high level of interpretation has an increased impact on decision-making (Trope and Liberman, 2010; Xu and Xie, 2011). In this study, in the process of weighing and evaluating risk options, decision-makers also follow this theory. That is, individuals in the proximal-space distance will give greater weight to the feasibility of the result, and those in the distant-space distance will give greater weight to the result. This ultimately leads to individual concerns about the feasibility of the result, and thus preference for risk avoidance in the proximal-space distance. On the contrary, the individual pursues the high value of the result and ignores the consideration of feasibility, and thus prefers risk-seeking in the distant-space distance.

In Study 2, it was also found that there is a significant interaction between the spatial distance and the decision framework. In the income framework, the long-space distance leads to more risk-seeking than the proximal-space distance, while the proximal-space distance leads to more risk aversion. In the loss framework, spatial distance has no effect on risk-seeking, and both proximal- and distant-space distance tend to be risk-seeking in the loss framework, and there is no significant difference between the two. This demonstrates that the influence of spatial distance on risky choice mainly exists in the acquisition framework. In addition, the classic framing effect is replicated under proximal-space distance conditions but eliminated under long-space distance conditions, which is more consistent with Raue et al. (2015). This may be because the long-space distance increases the psychological distance, leading to a decrease in emotional resonance (Keysar et al., 2012). It is more difficult to be processed by cognition, and the perception is not smooth, which will weaken one’s confidence in their judgment and make people more reliant on thoughtful thinking (Alter and Oppenheimer, 2008) instead of intuitive thinking. Furthermore, this distance will weaken or even eliminate the framing effect (Keysar et al., 2012). However, the results of this study are contrary to those of Duan et al. (2013) on spatial distance, who concluded that there is a framing effect in the long-space distance, while this effect is weakened in the proximal-space distance. This may be because the study adapted the gas explosion problem of Li et al. (2000) to manipulate the spatial distance. Regardless of how the spatial distance of the gas explosion in the mining disaster is manipulated, it is important for decision-makers who do not live in the mining area. It is said that all have a relatively long spatial and psychological distance, and for decision-makers proximal to the mining area, they all have a relatively proximal spatial and psychological distance. Therefore, this may cause confusion in the operation of the spatial distance.

In addition, the results also indicate that the subjects prefer risk aversion only under the conditions of close spatial distance and frame acquisition. Under the other three conditions, subjects prefer risk-seeking. This may be the difference between spatial distance and decision frame. The interaction leads to stronger risk-seeking among subjects (Trautmann and Van de Kuilen, 2012). The interaction between the framing effect and the psychological distance can be explained by integrating expectation theory and explanation level theory. The explanatory power of the two theories depends on the specific profit and loss assessment and other decision-making situations (Zhang et al., 2017). From the results of this study, it can be seen that when loss aversion and a high level of explanation occur at one or both of them, it will cause individuals to prefer risk-seeking in risky choice. In summary, our research results indicate that the formation of a decision not only depends on the framework of gains and losses but is also affected by spatial distance. The closeness or alienation of psychological distance may play an important role.

## Data Availability Statement

The raw data supporting the conclusions of this article will be made available by the authors, without undue reservation.

## Ethics Statement

The studies involving human participants were reviewed and approved by the Ethics Committee of School of Educational Sciences, Nanning Normal University. The patients/participants provided their written informed consent to participate in this study.

## Author Contributions

LH and FX: conceptualization. LH: data curation and formal analysis. FX: writing—original draft. Both authors contributed to the article and approved the submitted version.

## Conflict of Interest

The authors declare that the research was conducted in the absence of any commercial or financial relationships that could be construed as a potential conflict of interest.

## Publisher’s Note

All claims expressed in this article are solely those of the authors and do not necessarily represent those of their affiliated organizations, or those of the publisher, the editors and the reviewers. Any product that may be evaluated in this article, or claim that may be made by its manufacturer, is not guaranteed or endorsed by the publisher.

## Appendix 1

### Study 2 Risk Selection Questionnaire

During World War II, there were a large number of biological and chemical weapons in City H, which is 2,000/200 km away from your city. An accident caused a large number of these weapons to leak, which would cause the death of 1,000 Chinese residents. At that time, there were two options for controlling leaked weapons. The scientific evaluation of these two options is as follows. Which option do you support?

### Gain Frame

Plan A: If this plan is implemented, 400 people can be saved.

Plan B: If this plan is implemented, there is a 2/5 probability of saving 1,000 people, and a 3/5 probability of not saving anyone.

### Loss Frame

Plan A: If this plan is implemented, 600 people will die.

Plan B: If this plan is implemented, there is a 2/5 probability that no one will die, and a 3/5 probability that 1,000 people will die.
